# Health policy and systems research and analysis in Nigeria: examining health policymakers’ and researchers’ capacity assets, needs and perspectives in south-east Nigeria

**DOI:** 10.1186/s12961-016-0083-6

**Published:** 2016-02-24

**Authors:** Benjamin Uzochukwu, Chinyere Mbachu, Obinna Onwujekwe, Chinenye Okwuosa, Enyi Etiaba, Monica E. Nyström, Lucy Gilson

**Affiliations:** Department of Community Medicine, College of Medicine, University of Nigeria, Enugu-campus, Enugu, Nigeria; Department of Pharmacology and Therapeutics, College of Medicine, University of Nigeria, Enugu-campus, Enugu, Nigeria; Health Policy Research Group, College of Medicine, University of Nigeria, Enugu-campus, Enugu, Nigeria; Department of Health Administration and Management, College of Medicine, University of Nigeria, Enugu-campus, Enugu, Nigeria; Department of Learning, Informatics, Management and Ethics (LIME), Karolinska Institutet, SE 17177 Stockholm, Sweden; Department of Public Health and Clinical Medicine, Epidemiology and Global Health, Umeå University, SE90187 Umeå, Sweden; Health Policy and Systems Division, School of Public Health and Family Medicine, University of Cape Town, Cape Town, South Africa; Department of Global Health and Development, London School of Hygiene and Tropical Medicine, London, UK

**Keywords:** Capacity assets, Health policy and systems research and analysis, Nigeria

## Abstract

**Background:**

Health policy and systems research and analysis (HPSR+A) has been noted as central to health systems strengthening, yet the capacity for HPSR+A is limited in low- and middle-income countries. Building the capacity of African institutions, rather than relying on training provided in northern countries, is a more sustainable way of building the field in the continent. Recognising that there is insufficient information on African capacity to produce and use HPSR+A to inform interventions in capacity development, the Consortium for Health Policy and Systems Analysis in Africa (2011–2015) conducted a study with the aim to assess the capacity needs of its African partner institutions, including Nigeria, for HPSR+A. This paper provides new knowledge on health policy and systems research assets and needs of different stakeholders, and their perspectives on HPSR+A in Nigeria.

**Methods:**

This was a cross-sectional study conducted in the Enugu state, south-east Nigeria. It involved reviews and content analysis of relevant documents and interviews with organizations’ academic staff, policymakers and HPSR+A practitioners. The College of Medicine, University of Nigeria, Enugu campus (COMUNEC), was used as the case study and the HPSR+A capacity needs were assessed at the individual, unit and organizational levels. The HPSR+A capacity needs of the policy and research networks were also assessed.

**Results:**

For academicians, lack of awareness of the HPSR+A field and funding were identified as barriers to strengthening HPSR+A in Nigeria. Policymakers were not aware of the availability of research findings that could inform the policies they make nor where they could find them; they also appeared unwilling to go through the rigors of reading extensive research reports.

**Conclusion:**

There is a growing interest in HPSR+A as well as a demand for its teaching and, indeed, opportunities for building the field through research and teaching abound. However, there is a need to incorporate HPSR+A teaching and research at an early stage in student training. The need for capacity building for HPSR+A and teaching includes capacity building for human resources, provision and availability of academic materials and skills development on HPSR+A as well as for teaching. Suggested development concerns course accreditation, development of short courses, development and inclusion of HPSR+A teaching and research-specific training modules in school curricula for young researchers, training of young researchers and improving competence of existing researchers. Finally, we could leverage on existing administrative and financial governance mechanisms when establishing HPSR+A field building initiatives, including staff and organizational capacity developments and course development in HPSR+A.

## Background

Linking research for health with policies and decision making on healthcare in a country is necessary to provide decision-makers with empirically-based and scientifically-valid information on service delivery [1]. The importance of developing and investing on national health research capacity for local research institutes in low- and middle-income countries as a key element in the strengthening of these countries’ health systems has been emphasized globally [2]. Indeed, an important focus of the Bamako Call to Action was to ensure that research priorities were determined by countries, not global institutions [2]. In Africa, the number of skilled health researchers remains limited in relation to the burden of disease [3–5] and the 2013 World Health Report called for renewed efforts to strengthen health research capacity towards universal health coverage [6].

Health Policy and Systems Research (HPSR) has been defined as “*…the production of new knowledge to improve how societies organize themselves to achieve health goals*” [7]. HPSR aims to produce reliable and rigorous evidence which helps to inform the many and varied critical decisions that must be made by ministers of health, senior policymakers and health service managers on how to organize the various parts of the health system and effect desired changes [7]. HPSR has been noted as central to health systems development because it tries to draw a comprehensive picture of how the health system and broader determinants of health can shape and be shaped by policies. It therefore focuses primarily upon the more downstream aspects of health but does not address clinical management of patients or basic scientific research.

HPSR is a multidisciplinary and inter-disciplinary field and in which the people involved are of varied backgrounds and therefore varied needs. Some are trained social scientists with less understanding of the health sector, but with a wish to apply their skills to health systems questions, many come from broad public health backgrounds, with some experience in disease control programs, while others are clinical practitioners or researchers with limited exposure to social sciences [8, 9]. Given the diversity of individuals entering the field there is the potential risk for (1) a lack of clarity and common understanding of HPSRs scientific basis or (2) poor communication between disciplines [9, 10]. Both scenarios have negative consequences that can deter inter-disciplinary collaborations and result in an unstable and disunited field.

On the other hand HPSR and Analysis (HPSR+A), a term coined by the Consortium for Health Policy and Systems Analysis in Africa (CHEPSAA), embraces the full range of research and analysis relevant to health systems. This is important because, in addition to formal research, support for health system development typically includes routine analyses of information and analytic work conducted within policy environments. Given the importance of, and the need to strengthen research capacity in HPSR+A, this field is now receiving global attention [6, 9, 11, 12]. HPSR+A is an international priority and existing activity in Africa, but it is still an emerging field that needs support [8], and how most effectively to strengthen the capacity of the field remains clear [13].

In Nigeria, HPSR+A is still relatively new. However, it is a national priority and has been included in the National Strategic Health Development Plan as one of the eighth priority areas that aims to utilize research to inform policy and programmes, improve health and contribute to the global knowledge platform [8, 14]. Being a new area HPSR+A needs a lot of support to ensure that all health decisions are based on sound evidence. Currently, the use of research findings by policymakers and communities in Nigeria has been described as very limited, and a number of factors, including inadequate and insufficient capacity to produce and use HPSR+A, have been linked to this [15, 16].

Globally, it has been recognized that strengthening health systems is key to ensuring access to safe and effective health services for those most in need [17]. HPSR+A needs to be rooted in and responsive to national needs. Every country needs capacity to analyse its own health system and, drawing on international literature, develop and evaluate its own health system-strengthening strategies. Developing national capacity for HPSR+A is thus critical. The importance of HPSR+A in promoting evidence-based policymaking has therefore become very important, especially within the context of universal health coverage [6].

Generating appropriate, trustworthy evidence depends on the existence of good research organizations. At present, the capacity of such organizations in low- and middle-income countries is variable. Building the capacity of African institutions, rather than relying on northern training organizations, is a more sustainable way of building the field of HPSR+A in the continent [8]. Context-relevant capacity development interventions that are tailored to the needs of different entrants in HPSR+A and address the disciplinary diversity need to be developed while still maintaining the scientific foundations of HPSR+A [9, 13].

Investing in capacity development for local research institutes has also been emphasized in the policies of many international agencies [2, 18]. Furthermore, such local capacity is more likely to lead to local ownership of findings and the uptake of research evidence in policy decisions through closer connections between researchers and policymakers [19]. There have been few studies of the nature of capacity itself and even less in the area of the capacity of researchers and policymakers [20].

Designing a training program that meets the needs of target participants follows a sequence of steps that must start with the process of identifying capacity/competency requirements and the ‘gap’ between the kind of capacity that is required and what presently exits. Identifying this capacity ‘gap’ (which includes both existing and desired competencies and skills) is most imperative for the HPSR+A field, as it brings together people from a variety of backgrounds [21]. Recognising that there is insufficient information on African capacity to produce and use HPSR+A to inform interventions in capacity development, the CHEPSAA (2011–2015) conducted a study with the aim to assess the capacity needs for HPSR+A of the African partner institutions, including Nigeria. The objective of this paper is to provide new knowledge on HPSR+A assets and needs of different stakeholders and their perspectives on HPSR+A in Nigeria, and make recommendations for possible capacity development interventions. Local universities are central to strengthening HPSR+A capacity and CHEPSAA African partners already have capacity ‘assets’ to build upon.

## Methods

### Study area and design

This was a cross-sectional study conducted in Enugu state, south-east Nigeria. The College of Medicine, University of Nigeria, Enugu campus (COMUNEC) was used as the case study and the HPSR+A capacity needs were assessed at the individual, unit and organizational levels. The capacity needs of the college’s policy and research networks were also assessed, as well as the key policy and political contextual influences on the network member’s capacity to produce and use HPSR+A.

For the organizational capacity needs assessment, six leaders of COMUNEC were interviewed, specifically the Provost of the college, Deans of the three faculties, the college secretary and the financial controller (n = 6). For the unit assessment, the coordinators and staff of Health Policy and System units within the institution were interviewed, specifically the Health Policy Research Group (HPRG), the Department of Health Administration and Management, and the Department of Community Medicine (n = 3). While for the individual capacity assessment, a staff survey was conducted for 20% of COMUNEC staff selected by proportionate sampling.

A minimum sample size of 121 was calculated and approximated to 150, and the survey was sent to 150 staff with a response rate of 82%.

### Data collection and analysis

Data was collected in two phases. During the first phase, desk review of relevant national and local documents was done to capture the following themes: (1) decision makers capacity to use HPSR+A and its practical application; (3) major institutions involved in HPSR+A and teaching and critical mass of HPSR+A organizations, networks and their roles; (3) key policy and political facilitating and constraining factors that influence capacity to generate and use HPSR+A.

Review of documents was carried out between May and September 2011. Documents included in the review were existing and current information generated in COMUNEC’s daily activities on HPSR and analysis such as written regulations, vision and strategy documents, organizational charts, project and monitoring process documents, ethics approval documents, project proposals, spreadsheets of past and existing external funding, and project reports. Government documents related to health research capacity development, such as policy statements, strategic health plans and policy dissemination documents, were reviewed. Academic documents, such as published research papers on HPSR+A capacity and reports published to non-academic audiences, were also reviewed. In order to retrieve relevant institutional and government documents an extensive search was carried out on institutional and departmental websites (using Google search) and local libraries for grey literature. Academic databases such as PubMed and Cochrane were searched for published articles and reports. Different combinations of the following keywords were searched for on the websites: Nigerian, health, policy, system, research, capacity, needs, assets, institutions, teaching, quality, finance, leadership, governance, vision, funding. Expert recommendations and citation pearling were also used to identify and retrieve documents.

Once the documents were identified, the executive summary/abstract of each document was reviewed to identify its relevance to the study. The inclusion of documents in the review was guided by content of any of the aforementioned themes, their perceived relevance to HPSR+A capacity in Nigeria and availability of the document during the timing of the review. Relevant information on HPSR+A leadership and governance, HPSR+A teaching, health policy and system research currently undertaken and research quality assurance, demand for HPSR+A, HPSR+A communication and networking, and resources for HPSR+A.

During the second phase of data collection, both qualitative and quantitative data collection methods were used. In-depth interviews and focus group discussions were conducted for the key stakeholders identified, including leaders of HPSR+A institutions, administrators of HPSR+A institutes, staff involved in HPSR+A related projects, other generators of HPSR+A and users of HPSR+A (including students and decision makers), and key funders of HPSR+A and formalized networks (n = 25). The interview guides were adapted to different stakeholders to capture relevant information. A semi-structured questionnaire was used to conduct the staff survey.

Purposive sampling was used to identify key informants for in-depth interviews. The criteria for selection were leadership within COMUNEC and leadership of health policy and systems activities, staff or students engaged in HPSR+A and teaching within COMUNEC, staff engaged in HPSR+A in external organizations, government officials and policymakers mandated to develop health policies, plans and strategies within the ministry of health, key funders of HPSR+A and related health projects, or formalized health networks.

A total of 23 one-on-one interviews and two focus group in-depth discussions were conducted. Each interview was conducted by a moderator and a note-taker. The moderators were trained research fellows or assistants who are experienced in conducting in-depth interviews and were specifically trained to collect data for this study. The one-on-one interviews were conducted in the private offices of the participants or at an agreed-on convenient venue. The focus group participants were invited to the research office of HPRG in COMUNEC, where the discussions were held. Each group discussions had six participants and lasted an average of 43 minutes. The one-on-one interviews lasted an average of 27 minutes. With the consent of the participants, all interviews were audio-recorded using digital voice recorders.

In addition, a one-day needs assessment feedback workshop was held. The participants were grouped homogenously into (1) leaders of COMUNEC; (2) researchers in COMUNEC and other networks; (3) bureaucrats from the state ministry of health and politicians involved in policy formulation; (4) teachers of HPSR+A in COMUEC; (5) development partners and funders of HPSR+A; and (6) students in HPSR+A-related fields. They were asked to consider in what ways the findings have or not reflected the true picture. They were also asked about their reflections on building the field of HPSR+A in COMUNEC and its policy and research networks in Nigeria in terms of (1) what they would want the HPSR+A field to look like in COMUNEC, Enugu state and Nigeria, and what contributions they would make to achieve the vision; (2) who should be part of the field, who they would want to network with, and what role the networks would play in getting research into policy and practice and building the field of HPSR+A; and (3) what key competencies would be needed by people in the field – trainers, researchers, practitioners, policymakers, advocates, etc.

There are many different frameworks that help to conceptualize the different aspects of organizational capacity [22, 23], which often share some common features. HPSR+A capacity was therefore described in terms of:

1. Organizational leadership and governance in HPSR+A
2. Money and material resources for HPSR+A
3. Staff teaching expertise and skills in HPSR+A-related fields
4. Staff HPSR+A research skills, including research communication and networking skills
5. Decision-makers demand for and uptake of HPSR+A

Principal content analysis [24] was applied for qualitative data analysis. The interviews were transcribed verbatim and summarized to bring out the key points in thematic areas. The points were coded and similar points were aggregated and analyzed. Descriptive analysis was done for quantitative data using SPSS version 17. Open-ended questions were coded and descriptive statistics alone was calculated. Findings from the literature review, qualitative interviews and staff survey were triangulated [25].

Ethical approval was obtained from the Health Research Ethics Committee of the University of Nigeria Teaching Hospital, Enugu State. Informed consent was obtained from the respondents before data collection. At the end of the study, a feedback workshop was held for the policymakers in Enugu State and the academic staff from COMUNEC.

## Results and discussion

The findings from the study have been structured into four themes as follows:

- Key contextual influences on capacity to produce and use HPSR+A, including (1) government and organizational policies/plans/strategies that support HPSR+A; (2) political environment for HPSR+A; and (3) resource influence (funding and infrastructure)
- Capacity to generate HPSR+A in terms of (1) institutions involved in HPSR+A; (2) leadership and strategic vision for HPSR+A; and (3) staff HPSR+A teaching and research skills (assets and needs), including capacity to communicate HPSR+A
- Capacity to use HPSR+A in terms of (1) decision-makers demand for and uptake of HPSR+A and (2) their research uptake skills and its practical implications for evidence-based policymaking
- Groups’ feedback reflections

### Key contextual influences on generation and uptake of HPSR+A

#### Government and organizational policies/plans/strategies to support HPSR+A

The draft National Health Research policy, which was produced in 2001, provided some guidance to health research priority setting based on previously established criteria that lend relevance to decision making and would enhance uptake of research in policymaking. However, this document has remained a draft for over 10 years and resulted in irrelevant policy research outputs, limiting the uptake of HPSR+A.

A collaborative project between the Federal Government of Nigeria, the International Development Research Centre, Canada, and the Canadian International Development Agency, aimed at facilitating the Federal Government of Nigeria achieve its commitment to health sector reform, supported evidence-based decision making in the primary healthcare system through improved stakeholder participation in research priority setting, process and uptake of outputs for decision making.

The Nigeria Evidence-based Health System Initiative was developed as a 2-year extensive planning phase (2005–2007) to inform the implementation of a 6-year initiative (beginning in 2008) to support a fair, effective and efficient PHC through evidence-based decision making in two states in Nigeria: Bauchi and Cross River. The project aimed to strengthen local ownership of research output through involvement of stakeholders from the beginning of the process in identifying their own problems and priorities, and being involved in the collection, analysis and use of data to improve and make changes. The project builds on and complements the existing systems rather than setting up a parallel system. This initiative has been successful in the two pilot States.

The finalization of the National Strategic Health Development Plan in 2010, with details on interventions for evidence-based policymaking created an opportunity for research capacity strengthening, researcher-policymaker networking and discussions around HPSR+A [14]. Implementation of this plan has been slowed down by limited funding, resulting in poor capacity, particularly of implementers, to carry out HPSR+A and therefore low HPSR+A output.

The policy environment appears to have influenced the generation and uptake of HPSR+A in various ways. The development of policies and plans for health systems research shows some level of willingness to strengthen this field of research and probably inform better policy decisions, but the poor commitment to follow through with the plans hampers the materialization of any good will. The lack of implementation strategies and operational plans to strengthen the stewardship role of the government for research and knowledge management systems clearly constrains HPSR+A generation and uptake in Nigeria.

#### Political environment for HPSR+A

Lack of high quality research in Nigeria has been attributed to long periods of military rule that provided little or no support to research capacity development or its use for decision making, but was rather associated with high levels of emigration of qualified academics to the western countries. However, the return of civil rule ushered in a stronger role for research in policymaking by facilitating the inclusion of more academic and policy expertise into the policy process [26]. Uneke et al. [15] found, in their study on development of health policy and systems research in Nigeria, that at the organizational level, political interferences and influences constantly impede delivery of HPSR+A evidence and its use for decision making.

#### Funding and infrastructure for HPSR+A

The funding for HPSR+A teaching comes entirely from the government and is therefore sustainable. However, funding for research has been through external grants, which are limited to the length of the project and is therefore not sustainable. This was captured by an HPSR+A leader in the following quote:

“*Well, it is limited* […] *I know, but we are looking for funds; so we have some limitations. We have few sources and only two people are looking for the funds.*”

The annual health sector reform report for 2010 showed that the percentage of the health budget spent on health research and evaluation, and the proportion of research and evaluation studies undertaken on identified critical areas in the National Strategic Health Development Plan framework are yet to be determined [27]. However, the Federal Ministry of Health planned to spend 0.5% and 1% of the health budget on health research and evaluation by 2011 and 2013, respectively.

The funding streams for health research did not always complement organizational HPSR+A research priorities, with implications of poor or no uptake of outputs for decision making as exemplified by the following quote:

“*Some are, in fact the major ones we do are not, the funding source we have are not related to health systems and planning.*” (COMUNEC leader).

It was also noted that the organisation does not implement full cost recovery in making external grant applications since the rental cost of the premises is not paid for and the majority of the research staff are already employed by the University. Table 1 shows the key infrastructural challenges to HPSR+A within the organization.

**Table 1:** Proportion of staff with inadequate access to infrastructure

Variables	Proportion (n = 120), n (%)
Office space	62 (52.0)
Computers including internet and email	86 (72.0)
Electronic resources including online journals	95 (79.0)
Administrative and research specific software	106 (88.0)
Reliable electrical supply	100 (83.0)
Teaching space	84 (70.0)
Teaching equipment	89 (74.0)

The two major infrastructural challenges that influenced the generation of quality HPSR+A outputs were unavailability of research tools (software and e-journals) and poor electricity. Over 50% of staff did not have appropriate office space; on availability of research resources, 72% of the staff had neither computers nor internet facilities, 79% did not have electronic resources including online journals, 88% of administrative staff did not have administrative and research specific software; 83% of the staff did not have access to a reliable electrical supply since the alternative source of electricity (i.e. the generator sets) was often restricted to some offices to minimize cost.

### Capacity to generate HPSR+A outputs

#### Institutions involved in HPSR+A

There are four universities in Nigeria involved in both HPSR and teaching; the universities of Nigeria Enugu Campus, Ibadan, Maiduguri, and Ilorin. They are all public owned institutions and federal universities. The African Heritage Institution is a private institution comprised of researchers from different specialties who are also involved in HPSR+A research, but not in teaching.

University of Nigeria, Enugu Campus, has over 5 years of experience in post-graduate teaching of HPSR+A. In the Health Administration and Management Department, it is taught within the health systems and policy module for the award of the post-graduate diploma and MSc degree in Health economics, Management and Policy. In the HPRG, it is taught on ad-hoc basis to post-graduate public health residents doing their postings in the research group. It is also taught to research assistants and staff of COMUNEC in form of a continuing education programme. Furthermore, in the Department of Community Medicine, HPSR+A is taught within the research methodology and health management and policy modules for students pursuing a Master’s in Public Health degree.

The universities of Ibadan and Ilorin are located in south-west Nigeria. They offer diploma and master’s degree courses in health systems and planning within the Faculty of Public Health and Department of Community Medicine, respectively, where HPSR+A is taught as a module. The University of Maiduguri is located in north-east Nigeria and offers post-graduate training courses in health systems and planning.

#### Leadership and strategic vision for HPSR+A

There appears to be a well-established formal administrative structure and financial governance mechanisms that support good decision making in a hierarchical order, and with good staff representation.

**Fig. 1:**
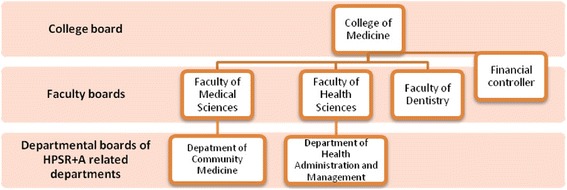
Hierarchical order of HPSR+A decision making within COMUNEC

As shown in Fig. 1, decision making for HPSR+A within the College of Medicine follows a hierarchical order. Within each department is a Departmental Board, which is made up of all the lecturers within the department and headed by the head of the department. Decisions within the departments are made by this board and are subject to approval by the Faculty Board, which is made up of representatives from each department. The chairman of the faculty board is the dean whose is elected by the faculty members every 2 years. Approvals from the Faculty board are subjected to the College Board, which consists of representatives of the different faculties and all Professors within the College. These representatives are elected by members of the faculty boards and headed by an elected chairman who is the provost.

While the leadership structure appears to be adequate and highly institutionalized, the vision for HPSR+A is not. There seems to be no shared understanding of HPSR+A in the institution. There are a few champions of HPSR+A who are actively involved in raising the profile of HPSR+A within and outside the organization, as reflected in this statement:

“*Yes. They* [HPSR+A champions] *are not injured or utilized by obstacles. They are not afraid of challenges. They are also accountable. They have vision of what is coming next* […] *and they network with so many partners.*” (Organizational leader)*.*

These champions exercise their influence through a multidisciplinary research group that reports on their undertaking of strategic HPSR+A and capacity development activities, and collaborates with policy and decision makers. However, it would appear that this small group of champions, for obvious reasons, have not been able to effectively translate their vision for HPSR+A to the wider organization.

The financial strategy, which is in place at the organizational and unit level to support HPSR+A, allows for diverse funding streams in seeking research grants. To ensure coordination of research funds and financial accountability, all grant approvals are currently managed by the office of the financial controller of COMUNEC.

#### Institutional and wider network HPSR+A capacity (assets and needs) including capacity to communicate HPSR+A

Out of the 71 academic staff that were surveyed, only four had any post-graduate training (5.6%), specifically MSc or PhD, in core HPSR+A fields, such as health economics, health services management/administration, health policy and planning, and health financing. Only two of the academic staff (2.8% or 50% of those with postgraduate training) had any teaching experience in HPSR+A. A total of nine academic staff (12.7%) had previous HPSR+A experience.

Table 2 below shows the capacity gap for skills required to carry out HPSR+A research among academic and non-academic staff of the institution. A majority of staff lacked capacity and skills in various areas of HPSR+A. A total of 101 staff (82%) lacked understanding of the concept of HPSR+A; over 60% of the staff needed skills in writing HPSR+A methodologies and dissemination outputs; over 75% needed leadership and networking skills for HPSR+A; 95 of the staff (77%) required skills in identifying and applying for grants; while 91 (74%) and 101 (82%) respondents lacked information management skills.

**Table 2:** Staff capacity need for HPSR+A

Capacity areas/skills for HPSR+A	Proportion of staff requiring additional training (n = 123), n (%)
HPSR+A technical skills and conceptual underpinnings,	
e.g. what is HPSR+A and what constitutes HPSR+A approaches to research and teaching	101 (82.0)
HPSR+A research and writing	
Writing research methodologies	84 (68.0)
Writing briefing notes for politicians, policymakers, external funders and donors	
	80 (65.0)
Writing papers for academic journals	78 (63.0)
Management and administration	
Leadership	92 (75.0)
Effective networking	94 (76.0)
Financial and information management	
Identifying and applying for external funding sources	95 (77.0)
Creating and managing effective and efficient financial reporting systems	91 (74.0)
Creating and managing effective internal information systems	101 (82.0)

Findings from the interviews support the survey result of poor staff capacity to assess grant opportunities. It showed that the institution as a whole has limited ability to apply for and obtain different funding streams since the number of staff that had training in grant application were very few.

“[…..] *There is also a very poor knowledge of how to obtain funds for research by the University Staff. They do not even know when these funds are made available.*” (Organization leader).

Responses from the interviews also corroborate with the survey findings of poor networking and information management capacity of staff of the institution. There are no established frameworks within COMUNEC that enable getting research into policy and practice (GRIPP) activities, though informal mechanisms have been used by the HPSR+A champions to involve stakeholders in research priority setting and process. As stated by a HPSR+A champion in COMUNEC:

“*We interact with these stakeholders by involving them in the projects from the onset* […] *we have informal and personal relationships with them.*” (HPSR+A champion).

Some respondents felt that while there appears to be good researcher-practitioner linkage, communication with policymakers is poor. One respondent also identified that the communication gap between researchers, donors and policymakers may explain the poor uptake of research outputs by policymakers:

“*There is also a communication problem between the donors and the researchers because the donors want a particular thing to be researched which might not be the crux of the problem.*” (Policymaker-bureaucrat)*.*

Existing channels of communication between researchers and policymakers that have been used by HPSR+A champions in the institution include briefing notes or policy briefs targeting policymakers, project reports for funders, dissemination workshops for academic and policy audience, technical meetings for bureaucrats, and academic publications aimed at other researchers and academics. Advocacy has been used as the most important means of strengthening linkages, and media and private consultancies as the means of engaging policymakers and practitioners.

Identified socio-cultural barriers for developing research-policymaker-practitioner relationships include poor understanding of research topics, corruption and office bureaucracy, national multi-ethnic and multi-tribal peculiarities that influence individual policymakers’ uptake of research evidence for decision making, and delay in dissemination of information. Some supporting quotes include:

“*We belong to a multi-ethnic, multi-tribe state so I think it affects researcher policymaker relationship in the sense that policymakers will accept those research findings that favour their people, they want to do what will benefit their people. He is going to ask first ‘is it my peoples problem or my ethnic group problem or my village problem’ before he gets involved.*” (Professional network)

“*Well one of the biggest socio-cultural barrier this organization has encountered in recent times is corruption; this has eaten deep into the bureaucratic principle of our society.*” (Bureaucrat)

A form of HPSR+A asset already exists among the policymakers. Out of the five policymakers interviewed, three had an MSc or MPH in core HPSR+A fields such as health economics, health services management/administration, health policy and planning, and health financing. One was a PhD candidate in health economics, management and policy, and the remaining one had no training in any of these fields. None had any teaching experience. The policymakers are also involved in research priority setting and process as they are engaged by the HPSR+A champions within COMUNEC. This has improved their capacity and it is a great asset.

### Capacity to use HPSR+A

The capacity to use HPSR+A is embedded in individual programmes that set their own capacity needs. Capacity constraints constitute the major challenge in the delivery of HPSR+A evidence in policymaking, and they include lack of access to information, poor capacity to collaborate with partners (e.g. researchers), lack of capacity development programmes, and inadequate funding.

#### Demand for and use of HPSR+A

Weaknesses exist in the utilization of evidence and decision making in the health sector has been described in previous research as strictly hierarchical, with a limited number of channels for innovation or implementation of research findings. The institutional roles for research coordination has been viewed as weak, with no frameworks for evidence-based decision making, and the frequent re-assignment of duties and roles discourages continuity of research projects and utilization of evidence for long-term planning and programming. As stated by some bureaucrats with regards to the mechanism for evidence-based policymaking:

“*We don’t have definite mechanism so to say* ‘*There is little or no research going on here.’ The ministry does not do so much of research and the much I have seen, research is completely dormant here and the main problem with the research is the funding.*” (Bureaucrats)

Findings from the interviews show a general perception that the demand for HPSR+A and its use in decision making is poor. Policymakers were seen not to value HPSR+A research because they lack understanding of research concepts, which added to the fact that the field of HPSR+A is relatively new. Variations noted across all the informants in their different levels of satisfaction with research output and its take up can be said to range from minimal to very low.

“*The policymakers in Nigeria do not have any idea of research in general and health policy research in particular so they don’t value it*” (Academia)*.*

“*Well the field of health policy and systems research is new here, even though there is need for much of the research, I don’t think that those who really need to request for it are even aware that it exists* [..].” (Policymaker-bureaucrat)

Almost all the respondents felt that research outputs and policy briefs are advertised but rarely used, one of the reasons being that they lack operational guidelines for policy implementation in line with recommendations.

“*As for policy briefs and their level of uptake, my job here is to make policies. Those briefs are there but are not comprehensive because they do not come up with operational guidelines of how the policy will be implemented or monitored.*” (Policymaker).

Another reason for poor uptake is limited access to research outputs, which was identified by a respondent and captured by the following quote:

“*There’s little or no demand for research outputs. Most research outputs end up in the shelves of various universities and institutions.*” (Academia)*.*

Policymakers in the Federal and State Governments and other health agencies have been said to be unwilling to initiate new research projects or plan services based on already existing research evidence since it is unlikely to be properly funded [28-30]. Such a situation affects the production of evidence-based research and compromises the quality of the research. If this becomes a vicious circle it will eventually result in a poor uptake of evidence in the entire health sector.

#### Decision-makers research uptake skills and its practical implication for evidence-based policymaking

One of the proposed reasons for poor uptake of research findings by policy and decision makers is their lack of research uptake skills due to their own education and training, which basically implies a lack of understanding of research outputs and low demand for high quality research [31]. The systemic barriers to research uptake that were identified include differences in timing of research outputs compared to when it is needed for policymaking. These results were also found in other studies [32]. Whereas researchers need as long as it takes to produce quality outputs, policymakers need clear findings at key points in the policy process, beyond which the outputs are irrelevant [32]. This implies that, if these outputs are not available at the point where they are needed, decisions will not be based on scientific and sound evidence. A supporting quote from the interview is:

“*Policymakers want quick answers, then researchers are too slow; they want to do it perfectly.* […..] *some policymakers are still used to non-evidence informed policymaking.*” (HPSR+A leader).

Another systemic barrier is the donor/funder-driven research priority. Because most researches are not government funded there may be distortions in priorities towards research that do not lend usefulness of evidence to decision making processes. Researchers may attempt to bridge this gap by seeking donor priorities that align with government priorities. However, because this rarely occurs, institutional research priorities are still majorly driven by available funding, as previously noted [32-34].

A major institutional barrier to research uptake, which inevitably affects decision makers’ research uptake skills, is the institutionalization of research and research uptake, or lack of it [32]. There exists a machinery to gather information at the state and federal ministries of health, through the Departments of Planning, Research and Statistics. However, the annual health sector reform report of 2010 identified that, although these Health Research Units exists, they are poorly staffed and underfunded [27]. There is no framework that details the processes and systems for research uptake activities for policymakers. This implies that there is no established process of accountability for decision making.

Individual contributors to decision-makers’ research uptake skills include the ability and capacity to (1) search for and access quality research outputs and (2) collaborate and form linkages with researchers [17]. Decision makers are of the opinion that research outputs are not readily available to them and that this hinders their use of this category of evidence for policymaking [15]. Uptake of training opportunities in HPSR+A by policymakers was also found to be poor, for reasons such as lack of awareness of local training institutes/opportunities, inconvenient mode of delivery (school-based teaching), and lack of interest.

“*Within the limits of the state, I am not conversant with a lot of policy teachings here*” (Policymaker-bureaucrat)

“*Well, I am quite aware that a form of teaching is going on but this is only at the University level.* […] *most people in the ministry* [Ministry of Health] *are just not interested no matter how much you teach them.*” (Policymaker-politician)

### Group feedback reflections

During the feedback workshop, there was a consensus that an accurate picture was captured by the study. Considering the fact that HPSR+A is a relatively new field, biomedical research still gets more attention from researchers and funders, and research uptake for decision making is generally poor in the state, with weak networks and no structure for GRIPP.

These findings generated some discussions around (1) how early and how much exposure to HPSR+A do students need to enable better understanding of the field, development of interest and increased participation in HPSR+A; (2) ways to institutionalize HPSR+A in the College of Medicine; (3) opportunities for collaborations within and between groups of actors in HPSR+A; and (4) ways of harnessing available expertise to overcome resource challenges. Some action points that were noted for future development in the institution and among networks included:

1. Need to build partnerships and networks between different groups of researchers in the University. It was agreed that the first point to start in building the field of HPSR+A is to identify the small groups of researchers and research units in the institution, and then create avenues for them to meet, exchange ideas, build interests and collaborate in researches.
2. Need for stronger presence and more visibility of the directorate for research and publication. It was noted that the directorate, which is a formal structure for coordinating research activities in COMUNEC, is not making enough impact because there is no framework in place for coordination of research activities. It was therefore agreed that (1) a framework for coordination of research activities needs to be developed, with significant inputs from HPRG; (2) a database of all groups involved in research needs to be set up by the directorate; (3) a functional accessible online library must be set up and a desk officer employed to manage it; (4) and scope and advertise calls for research proposals should be developed. It is hoped that this will attract researchers working in small groups to network and collaborate, to produce better quality responses to calls and higher quality research outputs.
3. Need to build researcher-policymaker networks with the State Ministry of Health.
4. Need to strengthen the capacity of policymakers in the State Ministry of Health to use research evidence for decision making as well as the capacity to contribute to the evidence generation process. To achieve this, it was suggested that the Ministry of Health needs to develop a research policy framework and implementation guidelines to enable proper research coordination and networking and the machinery for sustainability.

The groups’ reflections on building the field of HPSR+A in COMUNEC and its policy and research networks in Nigeria are shown in Table 3. In terms of vision, the HPSR+A, researchers and organization leaders felt that there should be a more formalized structure for HPSR+A and that researchers should drive the vision and the setting up of the policy and strategic plan for HPSR+A in Nigeria, with an identification of areas of HPSR+A needs and a clear budget envelope for HPSR+A. On the other hand, policymakers and development partners felt that there should be a robust evidence-based research network that can generate and communicate research findings and ideas to provide evidence for planning, decision making, and monitoring and evaluation of health system interventions. To be part of the field of HPSR+A, most participants were of the opinion that a wide range of stakeholders should be involved, including the academia, State Ministry of Health, researchers from other institutes, development partners and health professional organizations. Participants also felt that certain competencies are needed for the field of HPSR+A and teaching, including advocacy, information and communication technology skills, research methodology skills, skills in GRIPP, management skills, teaching/training skills, and mentorship skills (Table 3).

**Table 3:** Group reflections on building the field of HPSR+A in COMUNEC and its policy and research networks in Nigeria

Group of participant	Vision for HPSR+A and contribution to the vision	Who should be part of the field – possible networks and benefits	Key competencies needed
Researchers	Improved visibility, more representation and involvement of different fields, harmonization of stakeholders to ensure uptake of research findings, a formalized structure for HPSR+A, get all stakeholders involved in setting priority needs	Everybody is part of the network – university staff, staff in State Ministry of Health (SMOH), other health staff, other researchers from other institutes, donor agencies, beneficiaries, people from other fields with different expertise	Advocacy and communication skills (workshop, publications, etc.), research methodology skills, GRIPP skills, management skills, teaching/training skills
	HPSR+A researchers will drive the vision and setting up of the policy and strategic plan, share experiences of networking, be involved in training of other researchers and policymakers	This will bring in different perspectives, improve ownership and community participation, make for better adoption and priority setting and uptake	
Leaders in COMUNEC	A well-defined structure with a clear policy document, establish an office that will have a full-time employed research administrator (clearly defined roles – scouting for calls, publicizing calls, etc.) and budget for running the office must be available/provided by the college; harmonization of different research groups	Entire research team; networking with other research groups within and without the institution and country – important for capacity building	Expertise in the field, research interest/orientation, mentorship, computer literacy, workshop facilitation and training
Teachers of HPSA	Well-articulated HPSR+A structure with identification of areas of need in HPSR+A; a clear budget line for research within the institution that is managed by the directorate for research and policy	Whoever is willing and interested; networks with the university, development partners (e.g. WHO), donor agencies, professional groups (Nigeria Health Economics Association, African Health Economics and Policy Association, International Health Economics Association, etc.) There is a need to sensitize policymakers on the need to use evidence for policy, and through dissemination of research findings	
Bureaucrats and other policymakers	Contribute by developing research policy, implementation plan and budget, proper coordination and framework; ensure active participation; establish a researcher-policymaker network and machinery for sustainability; facilitate funding; carry out research gap analysis	SMOH, HPRG, Universities, development partners, private research organizations and private health practitioners; all tiers of government	HPSR+A research design, proposal writing and dissemination, data management packages, sourcing interpretation and utilization of research findings
Development partners and funders	A robust evidence-based research network that can generate and communicate research findings and ideas to provide evidence for planning, decision making, monitoring and evaluation of health system interventions	Development partners, Ministry of Health (all tiers), university community/researchers, private sector, civil society organizations, faith-based organizations, media	Analytical skills for policy analysis, negotiation skills, ICT skills, communication skills (presentation skills), training, technical skills
	Develop a research engine – i.e. a coordinating body to champion HPSR+A, with lobby groups that will cut across all groups of people and a robust research network that goes beyond COMUNEC	Networks should be developed to	
		o build a synergy among similar groups o knowledge management o resource mobilization and sharing o build capacity and confidence o provide robust and accessible database	

### Strengths and limitations of the study

The strengths of the study include the rigour of the methods used, mix of data sources and accessibility to a diversity of stakeholders. However, the paper covers only parts of Nigeria and may not adequately reflect the whole country. Despite the narrow coverage, the results may well impact health systems in other settings, particularly where similar situations are observed. There is need to explore what happens in other parts of the country.

## Conclusion and policy implications

Though funding was identified as a barrier to strengthening HPSR+A in Nigeria and at COMUNEC, a lack of awareness of the field was also a major constraining factor. The majority of the staff surveyed were not aware of what HPSR+A is or what it involves. All the academic staff surveyed were involved in some form of research, but due to a lack of awareness and motivation they were not concerned about policy or health systems implications and the impact of their research findings. Policymakers, on the other hand, were not aware of the availability of research findings that could inform their policy decisions, or where to find these research outputs, and were unwilling to go through the rigors of reading the research reports. It was observed that champions of HPSR+A research outside the College were the main sources of evidence for policymaking, although the institution had more academic researches and publications in journals.

Opportunities for strengthening HPSR+A and its teaching abound. There is a need to incorporate HPSR+A teaching and research at an early stage in student training. Departments that offer HPSR+A teaching and research need to be strengthened in terms of capacity building of human resources and availability of academic materials; course accreditation, development of short courses, and development and inclusion of HPSR teaching and research-specific training modules in school curriculum for young researchers; training of young researchers and improving capacity of existing ones; and capacity and skills development for HPSR+A research and teaching. Avenues for creating awareness of availability of research findings should be employed and collaboration with other HPSR+A research and teaching organizations and networks as well as collateral staff exchanges are available options. Advantage should also be taken of the already existing and adequate administrative and financial governance mechanism in establishing staff and organizational, as well as course, development in HPSR+A research and teaching.

Future engagement between policymakers and practitioners should include building the capacity of HPSR+A researchers in the areas of advocacy skills so that they can effectively determine the societal needs and the focus for policymakers; communication skills, especially where these relate to dissemination of HPSR+A research findings; and networking skills, including how to identify, build and sustain relevant networks. The further development and establishment of frameworks that enable networking and GRIPP activities within academic institutions, as well as, importantly, within policy institutions, is urgently needed, with consideration of socio-cultural peculiarities such as bureaucracy and information management. There is also a need to establish mechanisms for coordination of donors, research organizations and government in HPSR+A research priority setting. The identified existing channels of communication, specifically the use of briefing notes, dissemination workshops and technical meetings, need to be harnessed, as well as advocacy as a means of strengthening linkages and engaging policymakers and practitioners.

## References

[1] Global Health Council. Getting research into policy and practice (GRIPP). http://www.globalhealth.org/view_top.php3?id=186. Accessed 12 April 2014.

[2] World Health Organization (2008). Bamako Call to Action on Research for Health. Global Ministerial Forum on Research for Health.

[3] Whitworth JA, Kokwaro G, Kinyanjui S, Snewin VA, Tanner M, Walport M (2008). Strengthening capacity for health research in Africa. Lancet.

[4] Lazarus JV, Wallace SA, Liljestrand J (2010). Improving African health research capacity. Scand J Public Health.

[5] Ijsselmuiden C, Marais DL, Becerra-Posada F, Ghannem H (2012). Africa’s neglected area of human resources for health research - the way forward. S Afr Med J.

[6] World Health Organization (2013). World Health Report 2013: Research for Universal Health Coverage.

[7] Alliance for Health Policy and Systems Research. What is HPSR? 2007. http://www.who.int/alliance-hpsr/about/hpsr/en/index.html. Accessed on 12 March 2014.

[8] Mirzoev T, Lê G, Green A, Orgill M, Komba A, Esena RK (2014). Assessment of capacity for health policy and systems research and analysis in seven African universities: results from the CHEPSAA project. Health Policy Plan.

[9] Sheikh K, Gilson L, Agyepong IA, Hanson K, Ssengooba F (2011). Building the field of health policy and systems research: framing the questions. PLoS Med.

[10] Hoffman SJ, Røttingen JA, Bennet S, Lavis JN, Edge JS, Frenk J (2012). A review of conceptual barriers and opportunities facing health systems research to inform a strategy from the World Health Organization.

[11] Gilson L, Hanson K, Sheikh K, Agyepong IA, Ssengooba F (2011). Building the field of health policy and systems research: social science matters. PLoS Med.

[12] Bennett S, Agyepong IA, Sheikh K, Hanson K, Ssengooba F (2011). Building the field of health policy and systems research: an agenda for action. PLoS Med.

[13] Bennet S, Paina L, Kim C, Agyepong I, Chunharas S, McIntyre D, et al. What must be done to enhance capacity for health systems research? Background paper for the global symposium on health systems research. 2010. www.healthsystemsresearch.org/hsr2010/images/stories/4enhance_capacity.pdf. Accessed 15 April 2014.

[14] Federal Ministry of Health. National Strategic Health Development Plan 2010–2015. Abuja: Federal Government of Nigeria, ICF Macro, National Population Commission; 2010.

[15] Uneke CJ, Ezeoha AE, Ndukwe CD (2010). Development of health policy and systems research in Nigeria: lessons for developing countries’ evidence-based health policy making process and practice. Health Policy.

[16] COHRED. Health research in Nigeria – a summary. 2009. http://www.cohred.org/libarchive/content/661.pdf. Accessed 29 April 2014.

[17] Alliance for Health Policy and Systems Research, WHO. Sound choices: enhancing capacity for evidence‐informed health policy. 2007. http://www.who.int/alliance‐hpsr/resources/Alliance_BR.pdf. Accessed 14 April 2014.

[18] DFID (2008). DFID Research Strategy: 2008–2013.

[19] Innvaer S, Vist G, Trommald M, Oxman A (2002). Health policy-makers’ perceptions of their use of evidence: a systematic review. J Health Serv Res Policy.

[20] Nuyens Y (2007). 10 best resources for … health research capacity strengthening. Health Policy Plan.

[21] Walt G, Shiffman J, Schneider H, Murray SF, Brugha R, Gilson L (2008). ‘Doing’ health policy analysis; methodological and conceptual reflections and challenges. Health Policy Plan.

[22] Green A, Bennett S (2007). Sound choices: enhancing capacity for evidence-informed health policy.

[23] Lusthaus C, Adrien M-H, Perstinger M (1999). Capacity development: definitions, issues and implications for planning, monitoring and evaluation. Universalia Occassional Paper.

[24] Hsieh HF, Shannon SE (2005). Three approaches to qualitative content analysis. Qual Health Res.

[25] Thurmond VA (2001). The point of triangulation. J Nurs Scholarsh.

[26] Aberman N, Schiffer E, Johnson M, Oboh V. Mapping the policy process in Nigeria. Examining linkages between research and policy. NSSP Working paper. 2009. https://www.ifpri.org/publication/mapping-policy-process-nigeria. Accessed 15 April 2014.

[27] Federal Ministry of Health. Nigerian Annual Health Sector Report, 2010. Abuja: FMOH; 2011.

[28] Ajakaiye, O., Productivity and Capacity building in the Nigerian Research environment. NISER, Ibadan. http://www.cenbank.org/out/publications/occasionalpapers/rd/2000/ABE-00-3.PDF. Accessed 25 April 2013.

[29] Anofi D. Stakeholders decry non-passage of Health Bill. The Nation. http://www.thenationonlineng.net/2011/index.php/newsextra/4894-stakeholders-decry-non-passage-of-health-bill.html. Accessed 14 May 2014.

[30] Chiejina A. Shareholders worry over delay in National Health Bill passage. Business Day. 2011. http://chiejinaalex.blogspot.gr/2011/05/shareholders-worry-over-delay-in.html. Accessed 20 April 2014.

[31] Chiemeke S. Research outputs from Nigerian tertiary institutions: an empirical appraisal. Library Philosophy and Practice Journal. 2009. http://www.webpages.uidaho.edu/~mbolin/chiemeke-longe-shaif.htm. Accessed 14 May 2014.

[32] Knezovich J. The challenges of research uptake: systemic, institutional and individual barriers. Development research uptake in sub-Saharan Africa. 2012. http://www.futurehealthsystems.org/blog/2012/12/17/the-challenges-of-research-uptake-systemic-institutional-and.html. Accessed 15 April 2014.

[33] Egunjobi TO. Some aspects of the charm between social researchers and policy makers in Nigeria. 1981. http://www.cenbank.org/out/publications/occasionalpapers/rd/2000/abe-00-3.pdf. Accessed 29 April 2014.

[34] Nigeria Evidence-based Health System Initiative (NEHSI). Project implementation plan November 2007–March 2011. http://web.idrc.ca/uploads/user-S/12378217331NEHSI_Overview_-_March_2009.pdf. Accessed 10 April 2014.

